# RETRACTION NOTE

**DOI:** 10.3402/gha.v9.30899

**Published:** 2016-02-08

**Authors:** Giulia Ferrari

**Retraction:** Domestic violence and mental health: a cross-sectional survey of women seeking help from domestic violence support services.

Giulia Ferrari, Roxane Agnew-Davies, Jayne Bailey, Louise Howard, Emma Howarth, Tim J. Peters, Lynnmarie Sardinha, Gene Feder

**Retraction to:** Global Health Action (2014) 7: DOI: http://dx.doi.org/10.3402/gha.v7.25519

This article has been retracted by the authors following a revision of the numerical results. It is superseded by an article by the same title with the DOI: http://dx.doi.org/10.3402/gha.v9.29890

January 10, 2016 On behalf of the authors *Giulia Ferrari*

